# Extensive Gouty Tophus in Neglected Femoral Neck Fracture: A Case Report

**DOI:** 10.7759/cureus.61698

**Published:** 2024-06-04

**Authors:** Luis Henrique Longo, Bruno H Schuta Bodanese, Matheus U Senna Klipp, Luis Arthur C Colferai, Thayná C Silva, Rafael B Chruscinski, Ivan C Follmann

**Affiliations:** 1 Orthopaedics and Traumatology, Hospital do Trabalhador, Universidade Federal do Paraná, Curitiba, BRA; 2 Orthopaedics and Traumatology/Hip Surgery, Hospital Alemão Oswaldo Cruz, São Paulo, BRA; 3 Orthopaedics and Traumatology/Hip Surgery, Hospital do Trabalhador, Universidade Federal do Paraná, Curitiba, BRA

**Keywords:** total hip arthroplasty, gouty tophus, gout, neglected fracture, femoral neck fracture

## Abstract

Femoral neck fractures are extremely common injuries, especially in the elderly, who often have multiple associated comorbidities. Despite advances in surgical technique and implant technology, neglected fractures are still a reality in developing countries due to the lack of access to healthcare services or socioeconomic conditions of patients. This case report presents a 61-year-old male patient referred from a rural area to a trauma referral hospital with a neglected femoral neck fracture. The patient had multiple comorbidities, and during the surgical approach for total hip arthroplasty, the intraoperative finding of an extensive gouty tophus led to an increase in surgical time and modifications in the surgical procedure. The epidemiological profile of the patient in question fits the pattern of patients with diffuse gouty arthropathy, warranting suspicion of hip involvement when manifested in other joints. Performing complementary exams in patients preoperatively with proximal femur fractures and coxarthrosis can be an indispensable tool for the successful implementation of the therapeutic plan. This report presents these findings and the outcome of the method used.

## Introduction

Fractures of the proximal femur are extremely common injuries in elderly patients, usually preceded by low-energy traumas. This condition represents a challenge for physicians, patients, families, and society, given the high degree of morbidity and mortality associated with the condition, as well as the costs involved in the treatment and rehabilitation of these patients [1]. It is estimated that the United States alone has spent approximately 20 billion dollars per year (over the last decade) on managing this condition, where the prevalence may reach about 300,000 cases per year by 2030 [2]. 

Fractures of the femoral neck, in turn, represent a large portion of proximal femur fractures, corresponding to 40-50% of cases. It particularly affects White women over 65 years of age, with mobility impairments and low bone quality [3]. It is estimated that about 90% of elderly patients affected by femoral neck fractures also suffer from other comorbidities or polypharmacy, requiring a complex and multidisciplinary treatment approach. This indication reflects a mortality rate of up to 30% in the first year postoperatively [4]. 

Despite advances in the treatment of femoral neck fractures, such as implant quality, surgical technique, and patient rehabilitation, neglected fractures remain prevalent, especially in developing countries. The lack of access to healthcare services, as well as educational and economic factors, are some of the contributing factors to this reality [5].

This report aims to present the treatment experience of a patient admitted to our institution with a neglected femoral neck fracture, along with multiple comorbidities, associated with a distinct gouty lesion in the proximal femur. We present here the intraoperative findings and the outcome of the method used.

## Case presentation

A 61-year-old male patient, with a past medical history of systemic arterial hypertension, diabetes mellitus, dyslipidemia, and diffuse gouty arthritis, was referred from the countryside to a trauma reference service, with a report of a fall at ground level approximately six months ago, associated with chronic pain in the left hip and progressive worsening of gait. He reported previous independence in performing activities of daily living, deterioration of functional status since the traumatic episode, and currently being totally dependent on family members at the time of admission.

**Figure 1:**
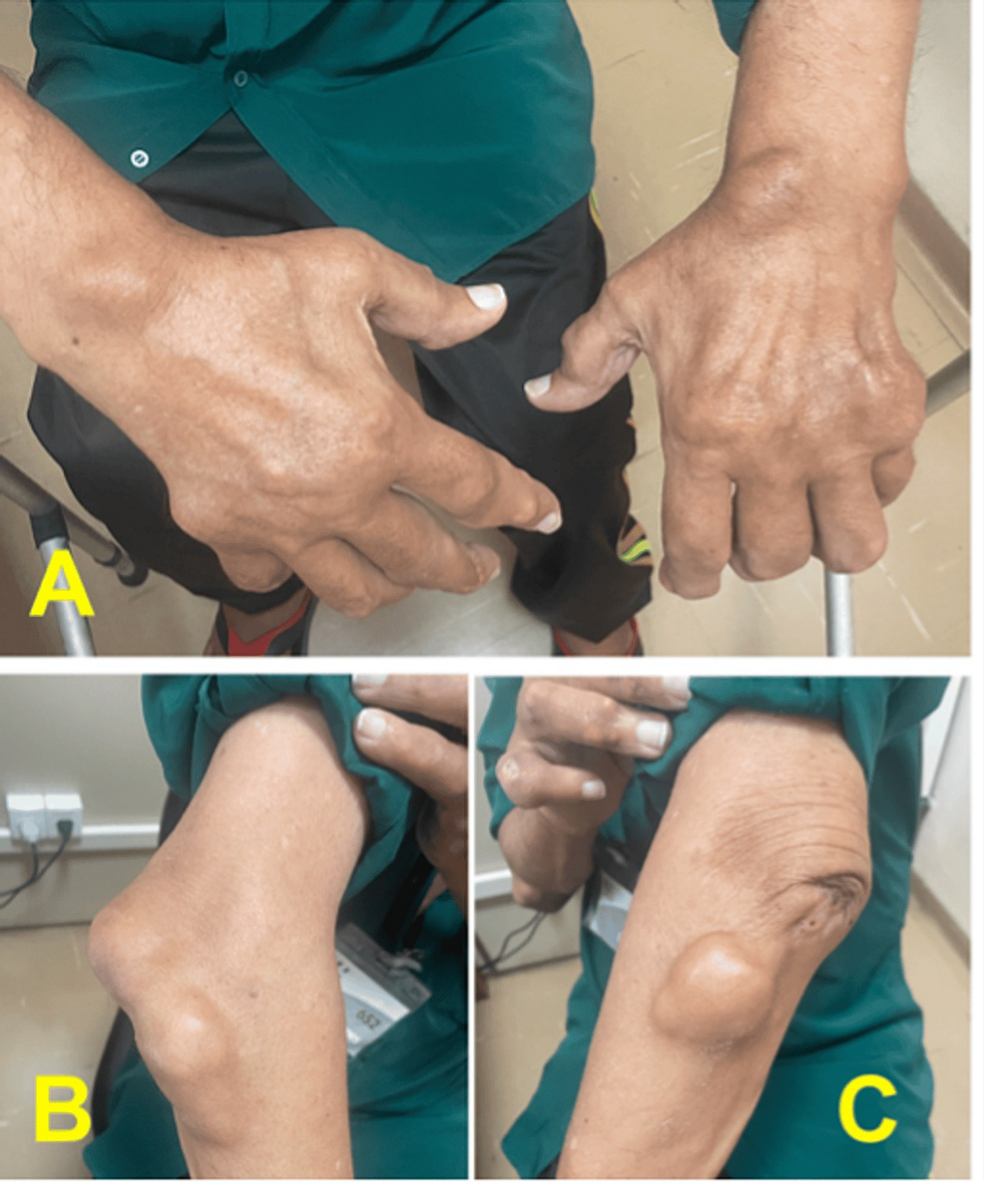
(A) Diffuse gouty tophi in phalanges, metacarpophalangeal joints, and wrists; (B) Presence of gouty lesions in the right elbow; (C) Gouty tophus in the left elbow

**Figure 2:**
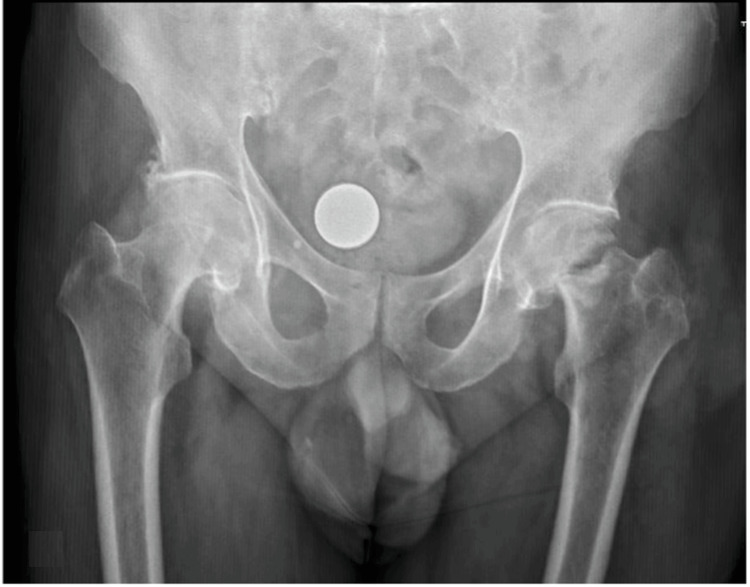
Anteroposterior pelvic radiograph, performed with traction and internal rotation of the limbs, showing an old left femoral neck fracture

To somatoscopy, the patient presented the left lower limb in external rotation, abducted, and slightly shortened; also noteworthy was the presence of diffuse gouty tophi in the joints (Figure 1). Radiographs in the anteroposterior (AP) of the pelvis were promptly performed, revealing a fracture of the left femoral neck (Garden 4 / AO 31B2.1) [6,7], with signs of sclerosis, well-defined contours, and bone resorption, suggestive of an old injury. These findings are represented in Figure 2.

Considering the patient's age, as well as the previous history of functional independence, definitive treatment with total hip arthroplasty on the left side was chosen, scheduled two days after hospital admission. After performing the surgical planning and complying with institutional safe surgery checklists, the posterolateral approach, also called the Kocher-Langenbeck approach, was chosen. 

**Figure 3:**
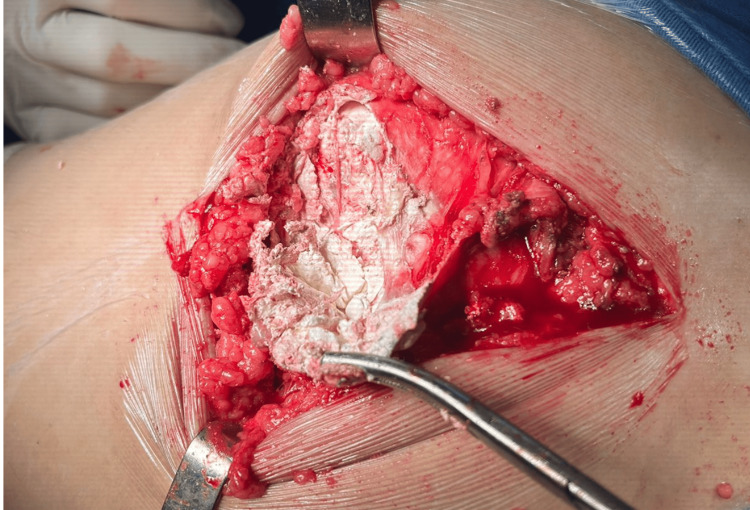
Gouty tophus occupying the incision area in posterolateral hip access

After the initial incision and dissection of the subcutaneous tissue, an extensive gouty tophus was observed in the pertrochanteric region; the lesion occupied practically the entire incision margin, approximately 10 cm long (Figure 3). The intraoperative finding required careful release of the lesion, which initially hindered the visualization of the fascia lata and iliotibial band. 

Subsequently, with the improvement of the visual and anatomical field, a longitudinal incision of the fascia lata and trochanteric bursectomy were performed, which soon revealed the continuity of the gouty lesion into the deep planes. As dissection progressed, the advancement of the gouty lesion towards the short external rotators and the joint capsule was noted. The extent of the tophus and its excision resulted in a considerable increase in surgical time, approximately 30 minutes. Additionally, there was a compromise of the local anatomy, given the inflammation and peripheral tissue fibrosis caused by the lesion.

**Figure 4:**
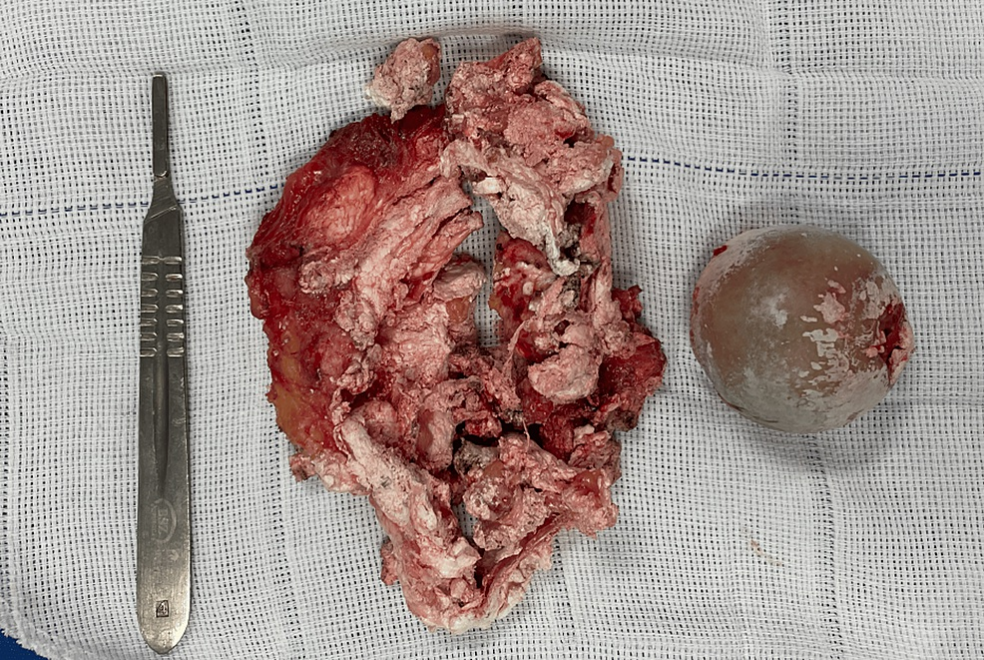
The aspect of the dried gouty tophus and devitalized femoral head

The femoral head osteotomy corroborated the observations from the preoperative radiograph: devitalized, with diffuse chondral wear, areas of resorption, and fibrosis (Figure 4). These findings, combined with the absence of a fracture hematoma in the joint capsule, point towards a chronic and neglected aspect of the fracture. The pathological study of the excised specimen confirmed the diagnosis of gouty tophus.

**Figure 5:**
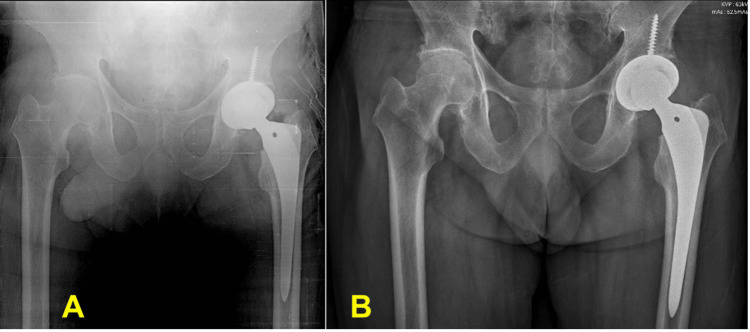
(A) Postoperative X-ray immediately after surgery; (B) Follow-up X-ray, with 12 weeks of progression.

Total hip arthroplasty was performed using the AESCULAP® Bicontact® implant (B. Braun, Melsungen, Germany), non-cemented, and proceeded without major complications (Figure 5). The neurovascular physical examination in the postoperative period was satisfactory, as well as the healing progression, with no signs of infection. Currently, the patient is being regularly monitored at the outpatient clinic and has resumed his activities independently. Additionally, the patient was also referred for optimization of gout clinical management with the hospital's rheumatology team.

## Discussion

It is estimated that the worldwide prevalence of gout is between 1-4% [8], with it being two to six times more common in men than in women. The annual incidence is about 2.68 per 1000 individuals. Gout is caused by hyperuricemia leading to urate deposition throughout the body and subsequent intermittent flares. Factors associated with lack of physical activity, consumption of processed foods, obesity, and metabolic syndrome contribute to the increased cases of gout [9]. Gout typically affects the first metatarsophalangeal joint (most common), but it can also involve joints of the midfoot, ankle, and upper limbs [10].

Although gout is the most common form of inflammatory arthritis, reports of detecting it in the hip joint are not common. The aging population, as well as the decline in renal function observed in elderly patients, contributes to increased uric acid accumulation in these patients. Hip surgeons, due to their greater contact with this patient profile, should be attentive to the possible complication of uric acid crystals in the joint [9]. The gold standard for diagnosing the disease is confirmation of monosodium urate in synovial fluid [11]. However, as reported in the present case, patients often do not present visible changes in the hip joint, so suspicion should arise for those with presentations in other joints and limbs. Previous studies report that bone erosions, sclerosis, decreased joint spaces, and tophus calcification can be observed on radiography, especially in well-established diseases [12], similar to the imaging findings of the patient in the current case. If there is suspicion of gout in a patient with a proximal femur fracture or coxarthrosis, we consider that performing complementary exams preoperatively, such as ultrasound or magnetic resonance imaging, may be useful for aiding in diagnosis and surgical planning.

Total hip arthroplasty as a definitive treatment for femoral neck fractures is widely supported in the literature, especially for neglected fractures with a given duration of evolution [5]. This type of implant favors the functional rehabilitation of the patient and the resumption of activities of daily living, being one of the most performed orthopedic procedures worldwide [13]. We believe that the prior diagnosis of a gouty tophus in the hip may modify the choice of approach for hip arthroplasty, depending on the anatomical involvement found and the safety of the surgeon. Moreover, introducing the debate about the adoption of a cementless stem in orthopedic procedures can certainly ignite heated discussions, particularly when considering elderly patients with systemic comorbidities, such as gout. Despite the possibility that the femoral canal may present anatomical and physiological characteristics that favor the use of this approach, it is crucial to emphasize the indispensability of longitudinal follow-up. Such a choice should not be taken hastily but rather be the subject of meticulous analysis, considering not only the individual peculiarities of the patient but also the potential challenges and advantages associated with the cementless fixation technique. This prolonged monitoring is imperative not only to track the patient's progress in the immediate postoperative period but also to early detect possible late complications, thus ensuring adequate clinical management and, consequently, optimizing surgical outcomes.

The association of multiple comorbidities in patients with femoral neck fractures, as well as socioeconomic conditions that determine therapeutic outcomes, are evidenced in the present study. Suspecting associated diseases that may interfere with the surgical procedure is crucial for optimizing operative time, controlling bleeding, therapeutic success, and patient prognosis.

## Conclusions

Femoral neck fractures are common injuries, often associated with other comorbidities in elderly patients. A thorough assessment of these is crucial for therapeutic success. Patients presenting with diffuse gouty arthropathy deserve careful attention to possible hip involvement, especially in preoperative femoral neck fractures or coxarthrosis. Investigation with complementary exams can not only contribute to the diagnosis of extensive gouty tophi but also lead to changes in the surgical plan.
